# Targeting pathogenic macrophages by the application of SHP-1 agonists reduces inflammation and alleviates pulmonary fibrosis

**DOI:** 10.1038/s41419-023-05876-z

**Published:** 2023-06-08

**Authors:** Shiao-Ya Hong, Ya-Ting Lu, Shih-Yu Chen, Chiung-Fang Hsu, Yi-Chun Lu, Cheng-Yi Wang, Kun-Lun Huang

**Affiliations:** 1 Department of Biotechnology and Laboratory Science in Medicine, National Yang Ming Chiao Tung University, Taipei, 11221 Taiwan; 2 Medical Research Center, Cardinal Tien Hospital, New Taipei, 23148 Taiwan; 3 Institute of Biomedical Sciences, Academia Sinica, Taipei, 11529 Taiwan; 4 Department of Internal Medicine, Cardinal Tien Hospital and School of Medicine, College of Medicine, Fu Jen Catholic University, New Taipei, 23148 Taiwan; 5 Graduate Institute of Medical Sciences, National Defense Medical Center, Taipei, 11490 Taiwan; 6 Division of Pulmonary and Critical Care Medicine, Tri-Service General Hospital, National Defense Medical Center, Taipei, 11490 Taiwan

**Keywords:** Cell death and immune response, Mechanisms of disease

## Abstract

Idiopathic pulmonary fibrosis is a progressive fibrotic disorder with no cure that is characterized by deterioration of lung function. Current FDA-approved drugs for IPF delay the decline in lung function, but neither reverse fibrosis nor significantly improve overall survival. SHP-1 deficiency results in hyperactive alveolar macrophages accumulating in the lung, which contribute to the induction of pulmonary fibrosis. Herein, we investigated whether employing a SHP-1 agonist ameliorates pulmonary fibrosis in a bleomycin-induced pulmonary fibrosis murine model. Histological examination and micro-computed tomography images showed that SHP-1 agonist treatment alleviates bleomycin-induced pulmonary fibrosis. Reduced alveolar hemorrhage, lung inflammation, and collagen deposition, as well as enhanced alveolar space, lung capacity, and improved overall survival were observed in mice administered the SHP-1 agonist. The percentage of macrophages collected from bronchoalveolar lavage fluid and circulating monocytes in bleomycin-instilled mice were also significantly reduced by SHP-1 agonist treatment, suggesting that the SHP-1 agonist may alleviate pulmonary fibrosis by targeting macrophages and reshaping the immunofibrotic niche. In human monocyte-derived macrophages, SHP-1 agonist treatment downregulated CSF1R expression and inactivated STAT3/NFκB signaling, culminating in inhibited macrophage survival and perturbed macrophage polarization. The expression of pro-fibrotic markers (e.g., *MRC1*, *CD200R1*, and *FN1*) by IL4/IL13-induced M2 macrophages that rely on CSF1R signaling for their fate-determination was restricted by SHP-1 agonist treatment. While M2-derived medium promoted the expression of fibroblast-to-myofibroblast transition markers (e.g., *ACTA2* and *COL3A1*), the application of SHP-1 agonist reversed the transition in a dose-dependent manner. Our report indicates that pharmacological activation of SHP-1 ameliorates pulmonary fibrosis via suppression of CSF1R signaling in macrophages, reduction of pathogenic macrophages, and the inhibition of fibroblast-to-myofibroblast transition. Our study thus identifies SHP-1 as a druggable target for the treatment of IPF, and suggests that the SHP-1 agonist may be developed as an anti-pulmonary fibrosis medication that both suppresses inflammation and restrains fibroblast-to-myofibroblast transition.

## Introduction

Idiopathic pulmonary fibrosis (IPF) is a rapid, progressive and lethal fibrotic disorder. It is characterized by dyspnea and progressive deterioration of lung function [1]. Two FDA approved small molecules, nintedanib and pirfenidone, have both been reported to show signs of slowing disease progression by reducing the decline in forced vital capacity (FVC) in clinical trials [2, 3]. However, neither drug can reverse the fibrosis nor significantly improve overall survival and the only other option is lung transplantation. A phosphodiesterase 4B inhibitor BI 1015550 has demonstrated promising results by restoring forced vital capacity, but more data is needed, including investigating concerns regarding vasculitis, to facilitate its approval [4]. Considering the increased burden associated with IPF, new therapeutic invention is urgently needed.

The precise role and involvement of the immune system in the pathogenesis of IPF remains largely elusive, but it provides a potent and early source of cytokines in response to tissue damage and repair. Recent studies have provided new insights into the roles of innate immune cells, particularly macrophages in pulmonary fibrosis. Macrophages represent the predominant population of immune cells in the lungs (constituting about 70% of the immune cells) and assume a crucial role in the process of airway remodeling in lung fibrosis [5, 6]. The polarization of macrophage is a fluid phenomenon in which macrophages exhibit diverse functional phenotypes in response to stimuli and signals from the microenvironment [7]. In the damaged tissue, macrophages can undergo polarization into distinct subsets of macrophages, categorized as classically activated (M1) and alternatively activated (M2) macrophages [8, 9]. It is believed that an excess of macrophage M1 polarization results in the demise of epithelial cell, while an uncontrolled polarization of M2 macrophages leads to the fibrotic remodeling of organs, including the lung [10, 11]. Recent reports even suggest that monocyte-derived alveolar macrophages are required for the development of pulmonary fibrosis [12–15]. A retrospective study also indicated that an increase in the monocyte count in the peripheral blood mononuclear cells (PBMCs) predicts a poor outcome in IPF patients [16]. Thus, targeting pathogenic macrophages has emerged as a strategy to limit inflammation during pulmonary fibrosis [17].

The Src homology domain 2 (SH2)-containing tyrosine phosphatase-1 (SHP-1; PTPN6) is a protein tyrosine phosphatase predominantly expressed at high levels in hematopoietic cells, at lower levels in most epithelial and some neuronal cells, with little or no expression in fibroblasts [18–22]. The binding of SH2 domains of SHP-1 and receptor pTyr residues leads to dephosphorylation of downstream JAK and/or STAT proteins [23]. Mice genetically lacking SHP-1 (*me* and *mev*) had a massive accumulation of hyperactive alveolar macrophages in the lungs, and displayed a profound susceptibility to naturally develop pulmonary fibrosis [24, 25]. The evidence suggested that SHP-1 might contribute to the restraint of aberrant innate immune cell activity, which assumes a pivotal role in the pathogenesis and progression of IPF. However, whether the application of SHP-1 agonists can restrain macrophages, reshape the immunofibrotic niche, and ameliorate IPF progression remain largely undetermined.

To explore the role of SHP-1 in the pathogenesis of IPF, SC-43, a small molecule SHP-1 agonist [26, 27] was administrated in bleomycin-induced pulmonary fibrosis mice, which are a broadly used pre-clinical model for IPF research. The therapeutic activities of SC-43 against pulmonary fibrosis and whether the SHP-1 agonist modulated macrophage homeostasis were investigated.

## Materials and methods

### Murine pulmonary fibrosis model

Female C57BL/6J mice (6–8 weeks old) were purchased from the National Laboratory Animal Center, Taipei, Taiwan. The murine pulmonary fibrosis model was established by delivering bleomycin solution to the lungs of mice with a MicroSprayer aerosolizer (IA-1C; Penn-Century, PA, USA) inserted into the trachea. Briefly, mice received a single dose of bleomycin (Bleocin, Nippon Kayaku, Japan) in 50 μl of sterile phosphate-buffered saline (PBS) by intratracheal spray under anesthetization. At day 7 after intratracheal spraying, the mice were randomized into three groups without blinding to receive vehicle, 60 mg/kg nintedanib, or 50 mg/kg SC-43 via oral gavage until the end of the experiment. Blood samples were obtained by cardiac puncture in EDTA microtainer tubes (BD Biosciences, NJ, USA) and sent to the National Laboratory Animal Center for complete blood count. The lungs were lavaged twice with 1 ml cold sterile saline through a plastic cannula. The bronchoalveolar lavage fluid (BALF) was centrifuged at 300 × *g*, 4 °C for 5 min. The cell pellets were resuspended in the cell staining buffer (2% FBS and 0.02% NaN3 in PBS) for flow cytometry analysis. All experimental procedures using these mice were performed according to protocols approved by the Institutional Laboratory Animal Care and Use Committee of Cardinal Tien Hospital (IACUC-108A-001 and IACUC-110C-005).

### Histochemical analysis

The lung samples were fixed in 4% buffered paraformaldehyde and embedded in paraffin. The paraffin embedded tissue array blocks were cut into 4-μm-thick sections for hematoxylin-eosin and Masson’s trichrome staining by the National Laboratory Animal Center (Taipei, Taiwan). Sections were scanned using the Pannoramic Desk Flash scanner and evaluated with Pannoramic viewer 1.15.4 software (3D HISTECH, Budapest, Hungary). An Ashcroft score was determined blindly to quantify the degree of fibrosis, according to a previously described method [28].

### Hydroxyproline assay

The amount of collagen in lung tissues was evaluated by measuring the levels of hydroxyproline using a total collagen assay kit (Biovision, CA, USA). The data were expressed as the collagen content per unit weight of lung tissue.

### Micro-computed tomography imaging

Mice were scanned in the supine position with SkyScan 1176 micro-computed tomography (CT) scanner (Bruker, Kontich, Belgium) at 7 and 21 days after bleomycin administration. Images were acquired throughout the spontaneous respiratory cycle with the following parameters: 17.76 μm pixel size, 0.5 mm Al filter, 50 kV source voltage, 500 μA source current, 280 ms exposure time. A total of 283 slices were reconstructed with NRecon software (version 1.7.1, Bruker). The frontal, sagittal, and transversal images were produced using the DataViewer software (version 1.5.1.2, Bruker). The rendered 3D models for virtual sections of all regions were created with the CTvox (version 3.0, Bruker). The lung volume was also determined by 3D reconstruction with CTvox, according to the previously described methods [29, 30].

### Flow cytometry

The cells were blocked with 1 µg anti-CD16/32 per 10^6^ cells for 15 min at 4 °C. Cells were incubated with antibodies in the dark for 1 h on ice. After washing with 1% BSA in PBS, cells were filtered and resuspended in the cell staining buffer for characterization of murine immune cell subsets according to a previously reported protocol [31]. The following antibodies were used: anti-CD3-BV650, anti-CD11b-Pacific Blue, anti-CD11c-PE/Cy7, anti-CD19-BV605, anti-CD45-Per-CP-Cy5.5, anti-MHC-II-BV510, anti-F4/80-PE, and anti-Ly6G-FITC (BioLegend, CA, USA). Data acquisition was performed on a CytoFLEX Flow Cytometer (Beckman Coulter, CA, USA) and analyzed with Kaluza software (version 4.2, Beckman Coulter).

### Cell culture and treatment

THP-1 and MRC-5 cells were procured from ATCC and underwent cytogenetic analysis for authentication by the established provider. THP-1 were cultured with RPMI 1640 medium supplemented with 2 mM L-glutamine, 1.5 g/l sodium bicarbonate, 4.5 g/l glucose, 10 mM HEPES and 1 mM sodium pyruvate, 0.05 mM 2-mercaptoethanol, 10% heat-inactivated fetal bovine serum. MRC-5 cells were grown in MEM media supplemented with L-glutamine, 10% fetal bovine serum and 1% Penicillin/Streptomycin. All cells were maintained at 37 °C in a humidified atmosphere of 5% CO_2_, and frequently tested for mycoplasma contamination using a commercially available EZPCR Mycoplasma Test Kit (Biological Industries, Beit-Haemek, Israel). THP-1 cells were differentiated into macrophages (M0) by 24 h incubation of 10 ng/ml Phorbol 12-myristate 13-acetate (PMA, Sigma-Aldrich, MO, USA) followed by a 72 h resting period. M0 macrophages were further polarized into M1 or M2 by treatment with IFNγ (20 ng/ml)/LPS (100 ng/ml) or IL-4 (20 ng/ml)/IL-13 (20 ng/ml) for 24 h.

### Cell viability and caspase-3 activity assay

Cell viability was measured using the AlamarBlue reagent (Invitrogen, CA, USA) and caspase-3 activity was performed using the Caspase-3 Colorimetric Assay Kit (BioVision, CA, USA) according to the manufacturer’s instructions.

### Immunoblotting

Total proteins were extracted with RIPA buffer (150 mM NaCl, 50 mM Tris-HCL, 0.1% SDS, 1% Triton X-100, 0.5% Na deoxycholate, 50 mM sodium fluoride, 2 mM EDTA) supplemented with 1× protease and phosphatase inhibitor cocktails (Roche Diagnostic, IN, USA). The antibodies used for immunoblotting against pY723-CSF1R (#3151), CSF1R (#3152), pY705-STAT3 (#9145), STAT3 (#9139), pS536-NFκB (#3033), NFκB (#8242), p-SMAD2/3 (#3108), SMAD2/3 (#8685), α-SMA (#19245) were obtained from Cell Signaling Technology (MA, USA), while antibodies against fibronectin (F3648, Sigma), collagen (ab6308, abcam), GAPDH (GTX100118, GeneTex), and β-actin (A4700, Sigma) were procured commercially. The images were acquired by the UVP ChemiDoc-It Imaging System (UVP, CA, USA).

### RNA extraction and reverse transcription-polymerase chain reaction

The RNA was extracted from cells using the GENEzol reagent (Geneaid, Taipei, Taiwan) and then reverse transcribed using the PrimeScript RT reagent Kit (Takara, Tokyo, Japan) according to the manufacturer’s protocol. Quantitative real-time PCR was performed on a Rotor-Gene Q real-time PCR system (Qiagen, Hilden, Germany) with the fluorescent dye SYBR Green methodology and analyzed using the manufacturer’s software. All primers in this study were synthesized by Integrated DNA Technologies (Singapore). Data were calculated by the 2^−ΔΔCt^ method, normalized to *GAPDH*.

### Sample preparation for single-cell mass cytometry

The sample were processed as described with some modifications [32–34]. Briefly, lung tissue was minced and enzymatically digested with 0.5 mg/ml collagenase type IV and 25 units/ml DNase I for 30 min at 37 °C. The resulting homogenate was filtered through a 70-µm cell strainer, centrifuged, and then resuspended in ACK lysing buffer (Thermo Fisher Scientific, MA, USA) for 5 min. The dissociated lung samples were washed and fixed with 1.5% paraformaldehyde at room temperature for 10 min, followed by two washes with cell staining media (CSM: PBS with 0.5% protease-free bovine serum albumin and 0.02% NaN3). The formaldehyde-fixed cell samples were then incubated with metal-conjugated antibodies against surface markers for 1 h at room temperature, washed once with CSM, permeabilized with methanol on ice for 10 min, washed twice with CSM, and then incubated with metal-conjugated antibodies against intracellular molecules for 1 h. The cells were washed once with CSM, and then incubated at room temperature for 20 min with an iridium-containing DNA intercalator (Fluidigm) in PBS containing 1.5% paraformaldehyde. After intercalation/fixation, the cell samples were washed once with CSM and twice with MilliQ water containing EQ Four Element Calibration Beads (Fluidigm) for normalization before measurement on a CyTOF mass cytometer (Fluidigm).

### Data acquisition and analysis for single-cell mass cytometry

Data preprocessing and analysis were performed as previously described [35]. Briefly, raw FCS files were normalized using the Fluidigm Helios software or the Premessa R package and gated in Cytobank (https://www.cytobank.org/). Doublets, debris and dead cell were gated out based on cell length, DNA content and cisplatin staining. Macrophages were defined by CD45+, CD3−, CD19−, Siglec-F−, Ter119−, Gr1−, Ly6G−, CD11b+ and F4/80+. The marker intensities were arcsinh transformed with a cofactor of 5, analyzed, and visualized using tSNE, FlowSOM and heatmap. tSNE maps were generated with software tools available at Cytobank by considering all markers except those used for gating.

### Statistical analysis

Mantel–Cox log-rank test was used to detect the difference in mouse survival. Statistical significance between groups was determined with one-way or two-way ANOVA followed by Bonferroni or Dunnett’s post hoc tests. The sample sizes were estimated from preliminary experiments and are indicated in the Figure legends. All data were expressed as means ± SD. Any *p* value of <0.05 was considered as statistically significant.

## Results

### SC-43 treatment alleviates bleomycin-induced pulmonary fibrosis

**Fig. 1:**
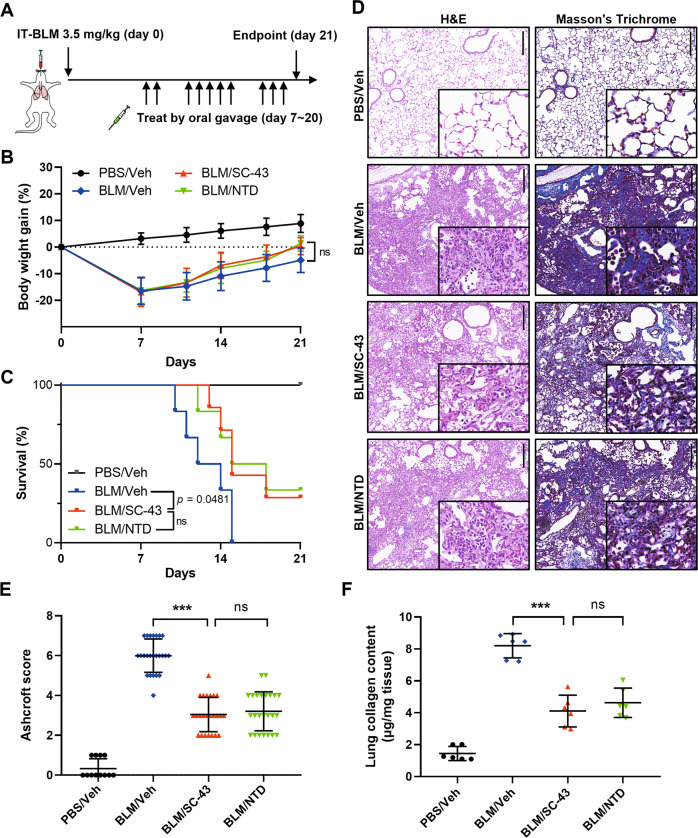
SC-43 treatment reduces alveolar hemorrhage, collagen deposition, inflammation, and improves the survival rate of bleomycin-induced IPF mice. **A** The scheme of experimental design. Mice receiving intratracheal instillation of 3.5 mg/kg bleomycin were administered vehicle, 50 mg/kg SC-43, or 60 mg/kg nintedanib (NTD) by gavage starting on day 7 until experimental or humane endpoints. Following bleomycin administration, body weight gain and survival rate were monitored over a 21-day period and lung tissues were assessed on day 21. **B**, **C** Body weight gain and survival rate of bleomycin-induced pulmonary fibrosis mice. **D** Representative images of lung tissue stained with H&E and Masson’s trichrome. Scale bar, 200 mm. **E** Quantification of lung fibrosis using the Ashcroft score. **F** Total collagen content in lung tissues determined by hydroxyproline assay. (*n* = 6 per group; ***p < 0.001).

In light of the role of SHP-1 in modulating IPF pathogenesis, the SHP-1 agonist SC-43 was used in the therapeutic bleomycin-induced mouse lung fibrosis model (Fig. 1A). Mice were intratracheally administered a high dose of bleomycin (3.5 mg/kg) (Fig. 1A). Seven days afterwards, mice were treated with vehicle, 50 mg/kg SC-43, or 60 mg/kg nintedanib (NTD) by daily oral gavage for 2 weeks. No significant differences in body weigh changes were detected between the bleomycin-treated mouse groups, irrespective of the treatment. The use of SC-43 or nintedanib did result in trends indicating improvement in body weight gain (Fig. 1B); however, these trends were not statistically significant. The analyses using the Mantel–Cox log rank test revealed that SC-43 administration significantly improved the survival rate in the bleomycin-induced pulmonary fibrosis model (*p* = 0.0481, Fig. 1C). As determined by hematoxylin and eosin staining of lung sections (Fig. 1D), bleomycin instillation resulted in impairment of normal lung architecture, infiltration of inflammatory cells, prominent accumulation of fibroblasts, and extensive deposition of fibrillar collagen. We found remarkable improvements following SC-43 treatment, such as reduced alveolar hemorrhage and inflammation, less collagen deposition and increased alveolar space. Ashcroft scores and hydroxyproline levels provided additional evidence of the improvements resulting from SC-43 treatment (Fig. 1E, F). Our findings suggest that SC-43 treatment offers protection comparable to nintedanib in terms of both survival rates and pulmonary fibrosis level (Fig. 1C–F).

**Fig. 2:**
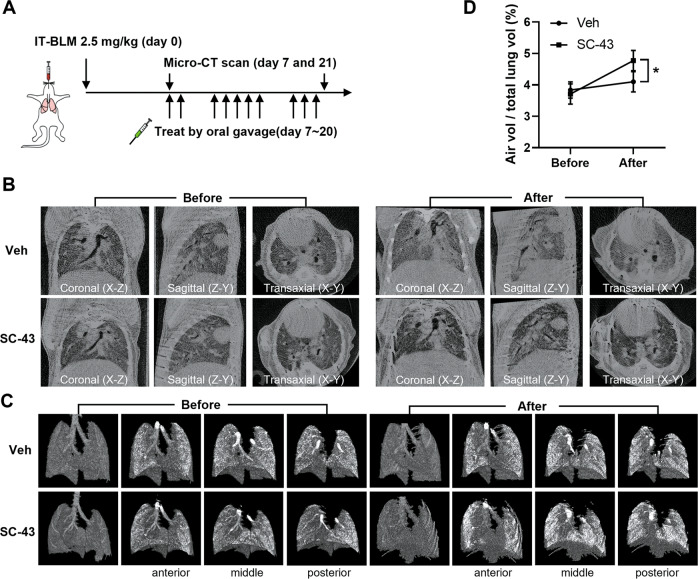
SC-43 treatment alleviates pulmonary fibrosis in bleomycin-induced IPF mice. **A** The scheme of experimental design. Mice receiving intratracheal instillation of 2.5 mg/kg bleomycin were administered by gavage on day 7 with vehicle or 50 mg/kg SC-43 for 2 weeks. Micro-CT images were acquired before and after treatment (day 7 and 21 after bleomycin injection) (**B**). The 3D X-ray dark-field radiographs (**C**) and air volume (**D**) of the same mouse were further analyzed by the CTVox software. Data are represented as mean ± SD and analyzed by two-way ANOVA with a Bonferroni post hoc test. (*n* = 3; *p < 0.05).

The therapeutic effects of SHP-1 agonist on air volume in the pulmonary fibrosis murine model were also investigated. Mice were intratracheally delivered with bleomycin (2.5 mg/kg), and SC-43 treatment was started 7 days later. The severity of pulmonary fibrosis and the alteration in air volume were examined by using micro-CT before and after the treatments (Fig. 2A). In the control group with vehicle treatment, micro-CT images revealed progressive anatomical changes of the lung architecture, including the distribution of ground-glass opacities and traction bronchiectasis. However, compared to the vehicle-treated group, SC-43 treatment reduced the pulmonary consolidation (gray area) (Fig. 2B). The topographical distribution of air volume was further visualized by reconstructed 3D images. Air volume of the lung was significantly recovered in SC-43-treated mice, compared to that in the vehicle group (Fig. 2C, D). The results showed that the SHP-1 agonist SC-43 actively exerts anti-fibrotic potency and ameliorates bleomycin-induced pulmonary fibrosis.

### SC-43 reduces bleomycin-stimulated macrophage accumulation in alveoli and rescues the neutrophil-to-lymphocyte ratio in peripheral blood

**Fig. 3:**
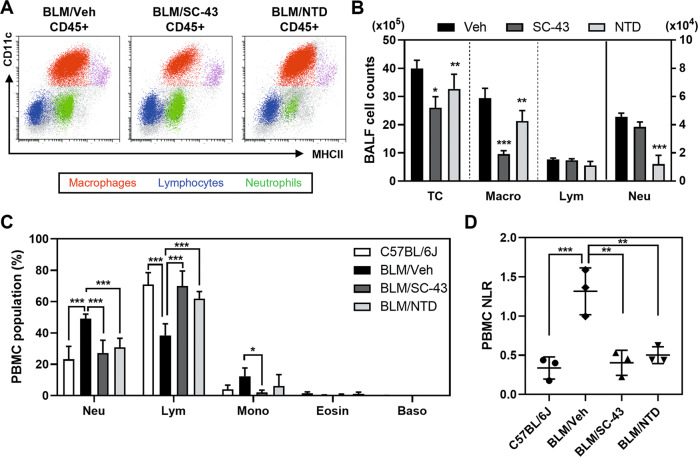
SC-43 treatment affects the distribution of immune cell population in IPF mice stimulated by bleomycin. Mice receiving intratracheal instillation of 2.5 mg/kg bleomycin were administered by gavage on day 7 with vehicle, 50 mg/kg SC-43, or 60 mg/kg nintedanib (NTD) for 2 weeks. All analyses were performed at day 21. **A** Representative plots of immune cell subsets in BALF. Macrophages, neutrophils, and lymphocytes are shown in red, green, and blue, respectively. **B** BALF cell differentiation was expressed as counts of total cells, macrophages, lymphocytes, and neutrophils per mouse lung. **C** The composition and proportion of neutrophils, lymphocytes, monocytes, and eosinophils in peripheral blood (PBMC). **D** The ratio of neutrophil to lymphocyte (NLR) in PBMC. (*n* = 3; *p < 0.05; **p < 0.01; ***p < 0.001).

As the immunomodulatory cells play a pivotal role orchestrating the immunofibrotic niche within the lung, we next sought to investigate whether the alleviation in pulmonary fibrosis following SHP-1 agonist treatment was a result of altered immune cell population. The bronchoalveolar lavage fluid (BALF) in bleomycin-induced pulmonary fibrosis mice was collected and the difference in immune cell population was analyzed. Phenotypic characterization of the BALF cells using multi-parameter flow cytometry showed that bleomycin induced a pronounced accumulation of macrophages (CD45^+^ CD11b^+^ CD11c^high^ F4/80^+^), neutrophils (CD45^+^ CD11b^+^ CD11c^low^ Ly6G^+^), and lymphocytes (CD45^+^ CD11b^−^ CD11c^low^) in the BALF on day 21 (Fig. 3A). Notably, a profound reduction in the numbers of alveolar macrophages, but not that of neutrophils and lymphocytes was observed in BALF of SC-43 treated mice (Fig. 3B). On the other hand, nintedanib exhibited a different profile by significantly reducing the numbers of neutrophils (Fig. 3B), and to a lesser extent, macrophages. The neutrophil-to-lymphocyte ratio (NLR) in the peripheral blood has emerged as a poor prognostic biomarker in various inflammatory diseases [36], and the high NLR in the peripheral blood of bleomycin-challenged mice was reversed by SC-43 treatment (Fig. 3C, D). Interestingly, SC-43 administration also decreased the proportion of circulating monocytes in the peripheral blood (Fig. 3C). These findings suggested that SC-43 may alter the immunofibrotic niche within the lung, as well as the systemic immune microenvironment, leading to the suppression of the pro-inflammatory responses required for the pathogenesis and progression of pulmonary fibrosis.

### SC-43 inhibits the CSF1R signaling axis in macrophages

**Fig. 4:**
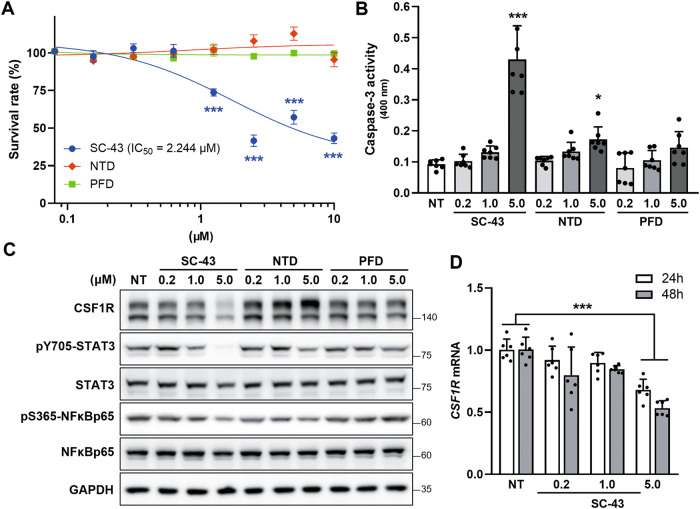
SC-43 targets macrophages through transcriptional downregulation of *CSF1R*. **A** Macrophage viability at indicated doses of SC-43, ninetadanib (NTD), and perfenidone (PFD). **B** Caspase-3 activity in SC-43, NTD, or PFD-treated macrophages. Values are expressed as mean ± SD of three independent experiments in duplicate and analyzed by one-way ANOVA with a Dunnett’s post hoc test. (***p < 0.001). **C** CSF1R expression and STAT3/NFκB activation after treatment with SC-43, NTD, or PFD. **D** *CSF1R* mRNA expression in macrophages after SC-43 treatment for 24 h and 48 h. Data are expressed as mean ± SD of three independent experiments in duplicates and analyzed by two-way ANOVA with a Dunnett’s post hoc test. (***p < 0.001).

As the results from the murine pulmonary fibrosis model showed that SC-43 reduced the numbers of macrophages in vivo, the effects of SC-43 on macrophage fate-determination were also evaluated in vitro. Human monocytic THP-1-derived macrophages were employed and incubated with the SHP-1 agonist SC-43. Compared to nintedanib and pirfenidone, which are the two currently approved medications for IPF, SC-43 treatment significantly reduced macrophage viability (Fig. 4A) by triggering macrophage apoptotic death (Fig. 4B). Notably, the expression of CSF1R and subsequently its downstream signaling (such as the phosphorylation of STAT3 and NFκB) were significantly downregulated in SC-43-treated, but not in nintedanib or pirfenidone-treated macrophages (Fig. 4C and Supplementary Fig. S1). Further analyses revealed that SC-43 treatment repressed the mRNA level of *CSF1R* in macrophages (Fig. 4D), while the proteasome inhibitor MG132, autophagic inhibitor chloroquine, and the lysosome inhibitor ammonium chloride failed to restore SC-43-induced CSF1R downregulation (Supplementary Fig. S2). The aforementioned results suggested that the SHP-1 agonist SC-43 may exert its anti-pulmonary fibrosis activities, at least partially, by transcriptionally reducing the expression of CSF1R in macrophages, which leads to the apoptosis of macrophages and reshaping of the immunofibrotic niche.

### SC-43 not only impairs the polarization of macrophages toward the pro-fibrotic M2 lineage but also alters M2 macrophage secretion, thereby preventing the fibroblast-to-myofibroblast transition

**Fig. 5:**
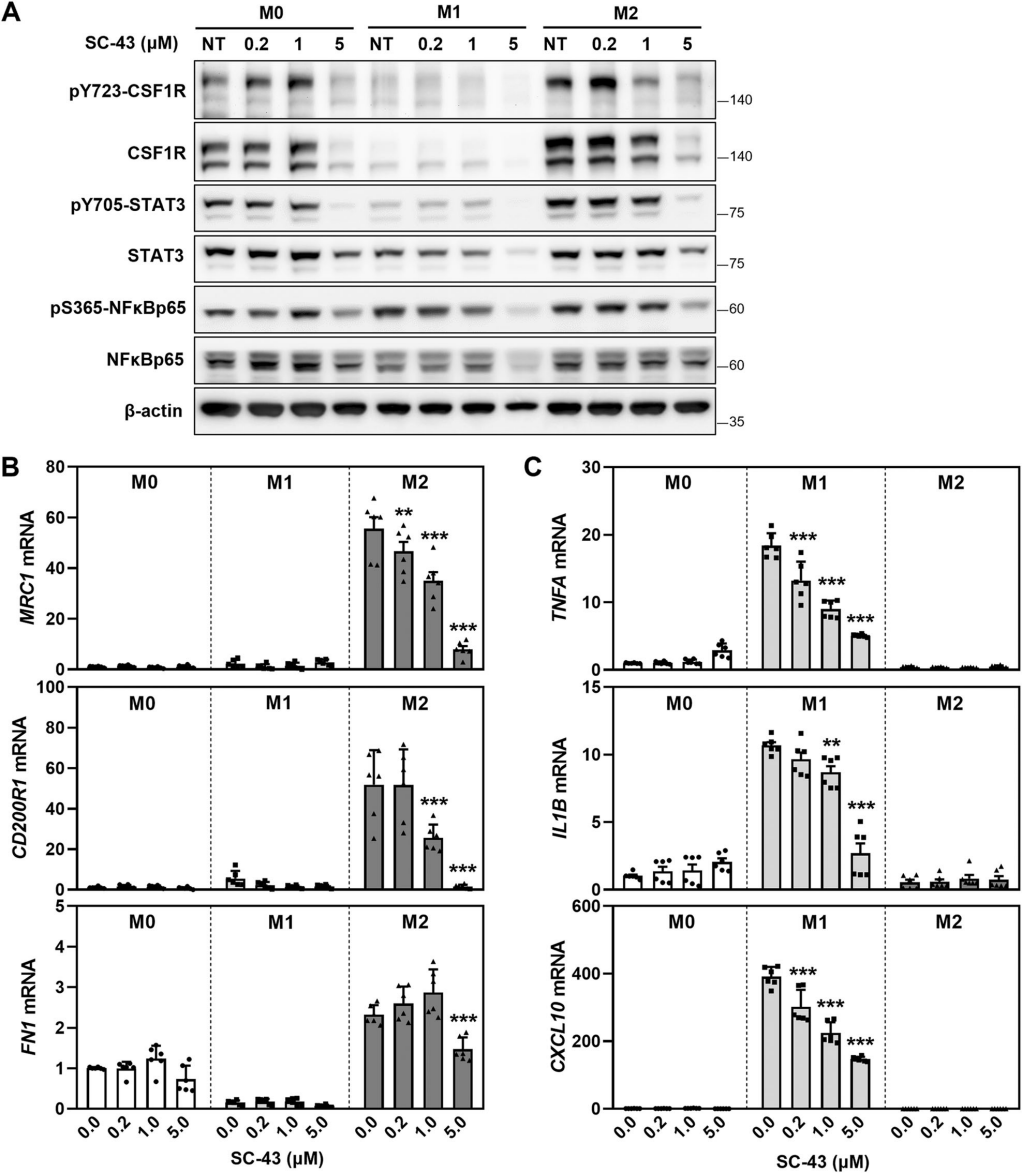
SC-43 downregulates the expression of pro-fibrotic genes in M2 macrophage and pro-inflammatory genes in M1 macrophages. **A** CSF1R/STAT3/NFκB signaling in SC-43-treated macrophages. The pro-fibrotic genes (*MRC1*, *CD200R1*, and *FN1*) of M2 (**B**) and the pro-inflammatory genes (*TNFA*, *IL1B*, and *CXCL10*) of M1 (**C**) were analyzed by RT-qPCR after SC-43 treatment for 24 h. Results are expressed as mean ± SD of three independent experiments in duplicates and analyzed by two-way ANOVA with a Dunnett’s post hoc test. (**p < 0.01; ***p < 0.001).

CSF1R signaling not only plays a critical role regulating survival, but has also been shown to direct macrophage polarization toward the pro-fibrotic M2 subtype, which is heavily involved in various models of fibrosis [17, 37–39]. Indeed, compared to macrophages with M1 phenotype, M2 macrophages expressed higher levels of total and activated CSF1R, which could be downregulated by SC-43 treatment (Fig. 5A). Increased expression of pro-fibrotic genes (*MRC1*, *CD200R1*, and *FN1*) was observed in IL4/IL13-induced macrophages (M2) compared to unstimulated macrophages (M0) or LPS/IFN-induced macrophages (M1) (Fig. 5B). The application of SHP-1 agonist SC-43 significantly reduced the expression of pro-fibrotic genes *MRC1*, *CD200R1*, and *FN1* in M2 macrophages (Fig. 5B). Following SC-43 treatment, the decreased expression of pro-inflammatory genes *TNFA*, *IL1B*, and *CXCL10* was observed in M1 macrophages, but not in M0 or M2 macrophages (Fig. 5C).

**Fig. 6:**
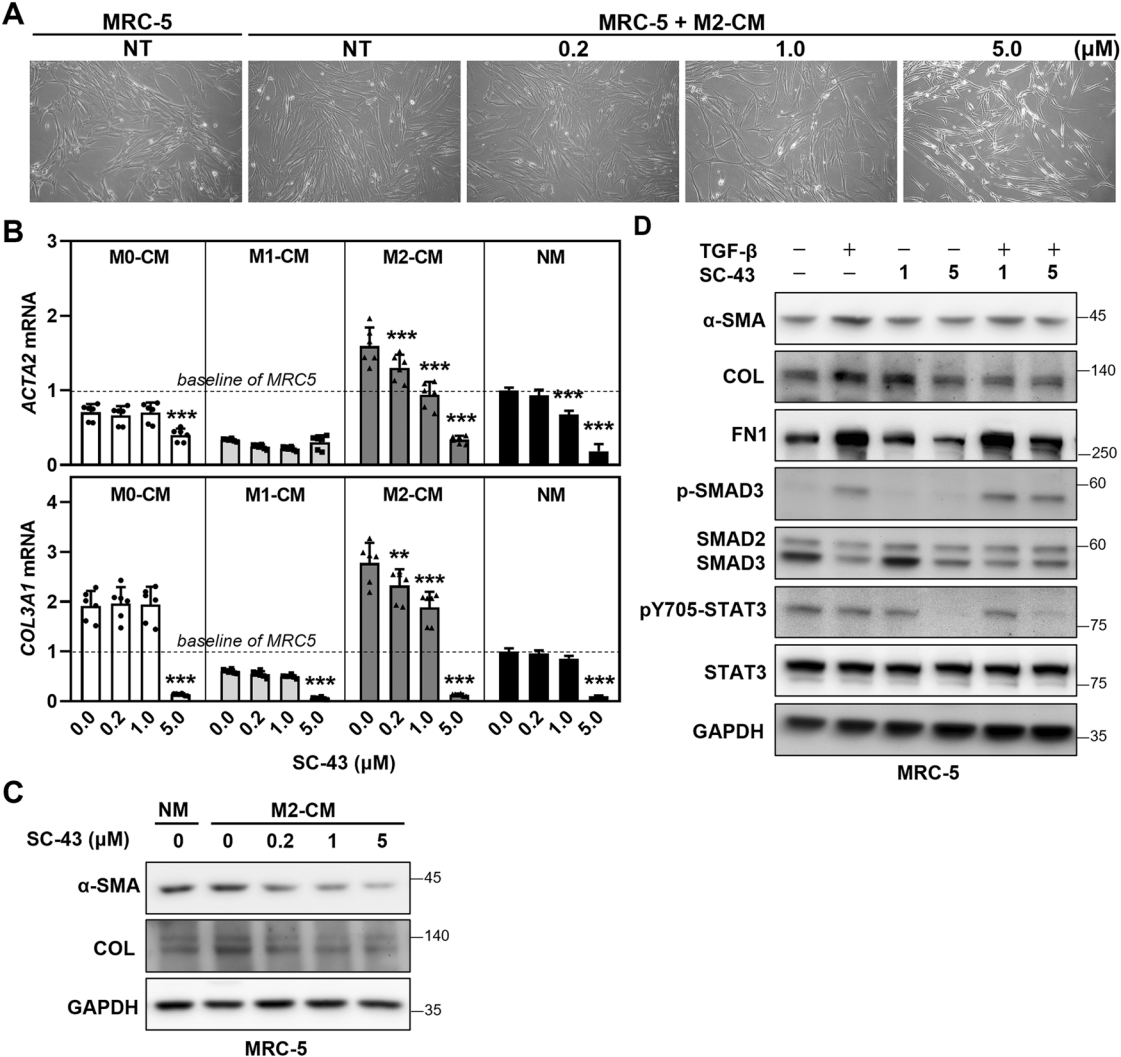
SC-43 treatment attenuates macrophage-mediated fibroblast-to-myofibroblast transition. **A** The morphology of MRC-5 fibroblasts stimulated for 48 h with CM derived from M2 macrophages. **B** The mRNA expression of α-SMA (*ACTA2*) and collagen (*COL3A1*) in MRC-5 fibroblasts. MRC-5 fibroblasts were stimulated for 48 h with CMs derived from untreated and SC-43-treated M0, M1, M2 macrophages, or with normal medium (NM). Results are normalized to parental fibroblasts, represented as mean ± SD of three independent experiments in duplicates, and analyzed by two-way ANOVA with a Dunnett’s post hoc test. (**p < 0.01; ***p < 0.001). **C** The expression of α-SMA and collagen in MRC-5 fibroblasts. MRC-5 fibroblasts were stimulated for 48 h with CMs derived from M2 macrophages treated with indicated doses of SC-43, or with normal medium (NM). **D** The effect of SC-43 on fibrotic protein expression in MRC-5 fibroblasts. MRC-5 fibroblasts were treated with indicated doses of SC-43 for 48 h.

Macrophages, especially the M2 subtype, secrete pro-fibrotic molecules to facilitate the transition of fibroblasts to myofibroblasts, which is a crucial cell population for collagen secretion and the pathogenesis of pulmonary fibrosis [40]. We thus examined whether SC-43 exerted its anti-pulmonary activities by altering the secretion of M2 macrophages and perturbing M2 macrophage crosstalk with the fibroblasts. M2-derived conditioned medium CM reprogramed the MRC-5 fibroblasts to acquire a myofibroblast-like morphology (Fig. 6A), and enhanced their mRNA levels of the pro-fibrotic α-SMA (*ACTA2*) and collagen (*COL3A1*) (Fig. 6B). Most importantly, CM collected from SC-43-treated M2 macrophages significantly attenuated MRC-5 fibroblast expression of fibrosis-related genes in a dose-dependent manner (Fig. 6B, C). Compared to the reduction in pro-fibrotic gene expression induced by adding 0.2 µM SC-43-treated M2-CM, no significant changes in MRC-5 fibroblasts’ pro-fibrotic gene expression were observed when normal media supplemented with 0.2 µM SC-43 was applied to MRC-5 fibroblasts. The results suggest that SC-43 may specifically exert its effects on M2 macrophage secretion, consequently inhibiting fibroblast-to-myofibroblast transition. On the other hand, 5 µM of SC-43 supplemented in normal media reduced the expression of pro-fibrotic genes in MRC-5 fibroblasts (Fig. 6B), indicating that SC-43, at a higher concentration, may directly affect fibroblasts and reduce their expression of pro-fibrotic genes. Notably, MRC-5 fibroblasts treated with 5 µM of SC-43 also exhibited decreased expression of FN1 under TGF-β stimulation (Fig. 6D).

### SC-43 selectively attenuates CSF1R/STAT3/NFkB signaling in alveolar macrophages following bleomycin stimulation

**Fig. 7:**
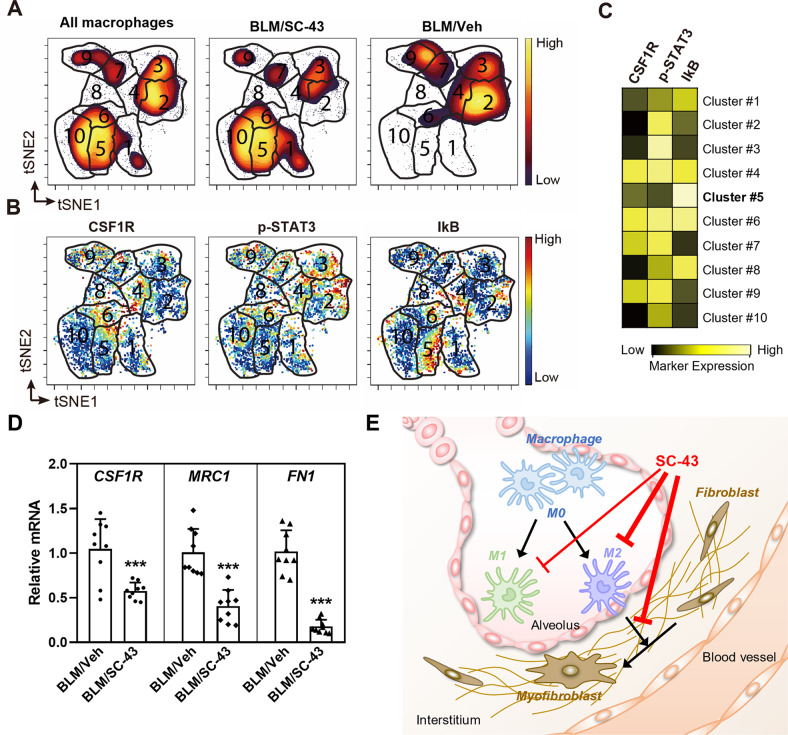
A small molecule SHP-1 agonist, SC-43 exerts an anti-pulmonary fibrosis activity by targeting macrophages. **A** The abundances of macrophages were visualized in tSNE maps, color-coded by cell density (yellow for high density, purple for low density). The number indicates the cluster ID of each FlowSOM metacluster. **B** The expression levels of the indicated markers visualized in tSNE maps. **C** Heatmap of median arcsinh-transformed intensities of indicated markers in each metacluster. **D** Fibrotic gene expression in BALF cells were detected using RT-qPCR. Results were normalized to IPF mice with vehicle and represented as mean ± SD of three mice in triplicates. The data were analyzed by two-way ANOVA with a Dunnett’s post hoc test. (**p < 0.01; ***p < 0.001). **E** This schematic diagram provides a brief overview showing that SC-43, via CSF1R downregulation, suppresses the survival and M2 polarization of macrophages, leading to the inhibition of fibroblast-to-myofibroblast transition and the alleviation of pulmonary fibrosis.

To elucidate the impact of the SHP-1 agonist on immune cells within the context of pulmonary fibrosis, we employed single-cell mass cytometry to analyze dissociated lung specimens from mice with bleomycin-induced pulmonary fibrosis, subjected to either SC-43 or vehicle-only treatment. A panel of 41 metal-conjugated antibodies (Supplementary Table S1), encompassing both lineage markers to define cell types and signaling molecules for functional assessment, was used to stain single-cell suspensions of lung samples. Our findings revealed distinct patterns in the macrophage compartment between the vehicle control and SC-43 treated animals, with a markedly enriched unique cell subset in the SC-43-treated group (cluster 5, Fig. 7A). This specific macrophage subpopulation displayed elevated IkB expression levels, while their phospho-STAT3 and CSF1R expression levels were diminished compared to other macrophage clusters (Fig. 7B, C). The results suggest that SC-43 treatment may potentially inhibit CSF1R expression, dephosphorylate STAT3, and inactivate NFkB pathways within macrophages. Furthermore, we obtained BALF cells from bleomycin-mice and conducted RT-qPCR to investigate the impact of SC-43 on fibrotic gene expression. We observed no significant alterations in the expression of pro-inflammatory markers *CD200R1*, *IL-1*, *TNFA*, and *CXCL10* on day 21 (data not shown). Nonetheless, the expression of *CSF1R*, *MRC1*, and *FN1* exhibited a significant reduction following SC-43 treatment (Fig. 7D), implying that SC-43 may inhibit the CSF1R/STAT3 signaling pathway and diminish the expression of fibrotic genes within macrophages in vivo during the pulmonary fibrosis progression. Collectively, our findings provide evidence for the pre-clinical efficacy of the SHP-1 agonist SC-43 in attenuating macrophage accumulation within the lung, mitigating pulmonary fibrosis, and enhancing survival.

## Discussion

Idiopathic pulmonary fibrosis (IPF) is a lethal and irreversible condition with no cure that is characterized by decline in lung function. As an excess of hyperactive alveolar macrophages and the tendency to develop pulmonary fibrosis is observed in SHP-1 deficient *me* and *mev* mice [24, 25], SHP-1 may be involved in macrophage homeostasis control and prohibiting the occurrence of pulmonary fibrosis. Our results indicated that SC-43 treatment reduces inflammation and collagen deposition in the lung (Fig. 1D), increases lung air volume (Fig. 2D), and most importantly improves survival rate (Fig. 1C) in the bleomycin-induced murine pulmonary fibrosis model. In addition, SC-43 not only reduces the macrophage population in BALF of bleomycin-induced mice, but also lowers the population of circulating monocytes in their peripheral blood. Decreased circulating monocytes as a result of SC-43 treatment against bleomycin-induced pulmonary fibrosis corroborates the efficacy of the SHP-1 agonist, as increased monocyte counts have been linked disease progression in IPF patients [41, 42]. At the molecular level, SC-43 downregulated CSF1R expression and its downstream STAT3/NFκB signaling in macrophages, thereby restricting macrophage M2 polarization and prohibiting M2-macrophage-orchestraed fibroblast-to-myofibroblast transition (Fig. 7E). To the best of our knowledge, this is the first proof-of-concept study in the pre-clinical setting demonstrating that pharmacological activation of SHP-1 effectively ameliorates pulmonary fibrosis. Our results suggest that the SHP-1 agonist SC-43, via targeting the CSF1R/STAT3/NFκB pathway in the alveolar macrophages, may hold great potential in pulmonary fibrosis resolution for IPF patients.

Several lines of evidence have indicated that IPF may be caused by persistent micro-injury and dysregulated repair of the alveolar epithelium [12]. Reports also suggested a role for M2 macrophage polarization in promoting pulmonary fibrosis. The fact that SC-43 reduced the expression of M2 markers (Fig. 5B), and CM collected from SC-43-treated M2 macrophages prohibited fibroblast-to-myofibroblast transition (Fig. 6B, C) highlights the role of M2 macrophages in the pathogenesis of pulmonary fibrosis. Interestingly, CM from M0 and M1 cells did slightly reduce the expression of *ACTA2* and *COL3A1* (Fig. 6B). However, most of the expression levels were below the baseline values of control fibroblasts (horizontal dash line, Fig. 6B); the effects of M2 macrophages on fibroblast-to-myofibroblast transition may be more prominent. In addition, fibronectin produced by alveolar macrophages has been linked to the recruitment of fibroblasts to injured tissue [43]. SC-43 treatment decreased *FN1* expression in M2 macrophages (Fig. 5B) suggesting another possibility, i.e., that SC-43 may contribute to mitigation of pulmonary fibrosis by inhibiting fibroblast recruitment. On the other hand, systemic increase of activity of TNF-α and IL-1β, which are markers of M1 macrophages, has been shown to promote myofibroblast differentiation and exacerbate bleomycin-induced pulmonary fibrosis [44–46]. Although the role of CXCL10 remains controversial, with studies suggesting that it has both pro- and anti-fibrotic functions [47, 48], CXCL10 has been shown to promote epithelial-to-mesenchymal and macrophage-to-myofibroblast transition in kidney fibrosis [49, 50]. Our results showed that SC-43 suppresses the pro-inflammatory TNF-α, IL-1β, and CXCL10 levels in M1 macrophages (Fig. 5C), indicating that SC-43 assumes an anti-inflammation role by inhibiting pro-inflammatory mediators, potentially relieving the early inflammatory phase of bleomycin-induced pulmonary fibrosis [51]. Therefore, SC-43 may be developed as an anti-fibrotic therapy for treating pulmonary fibrosis by assuming dual roles; first by suppressing early inflammation, and second via prohibiting the macrophage-induced fibroblast-to-myofibroblast transition during the progression of pulmonary fibrosis.

SHP-1 has been demonstrated to be a crucial negative regulator of pro-inflammatory cytokine signaling, TLR signaling, and inflammatory gene expression [52, 53]. However, the analysis of IPF patient samples sourced from the Gene Expression Omnibus database (GSE24206 and GSE2052) revealed no significant changes in SHP-1 expression (Supplementary Fig. S3). The multiple kinase inhibitor nintedanib, one of the two drugs currently approved for treating IPF, has been reported to enhance SHP-1 activity, leading to the dephosphorylation of pro-apoptotic factors and eventually inducing apoptosis of chronic lymphocytic leukemia cells [54]. Moreover, nintedanib can act as a SHP-1 agonist to inhibit STAT3/NFκB signaling [55] and has recently been reported to alter the phenotype of macrophages by inhibiting CSF1-induced CSF1R activation in vitro [56]. Sorafenib has also been found to enhance phosphatase activity of SHP-1 [57]. In mouse models of liver fibrosis, sorafenib ameliorates liver fibrosis by activating SHP-1 and inhibiting STAT3 to promote apoptosis of hepatic stellate cells [58]. SC-43, which has been reported to be a stronger SHP-1 agonist than sorafenib and binds directly to SHP-1 [26], exhibited activities against liver fibrosis [27]. However, whether the SHP-1 agonist SC-43 could be used to resolve pulmonary fibrosis has never been investigated. Our current study showed that the small molecule of SHP-1 agonist SC-43 downregulates CSF1R and inhibits STAT3/NFκB signaling pathways, which in turn modulates macrophage survival/polarization, leading to suppressed fibroblast-to-myofibroblast transition, reduced collagen deposition, and alleviated pulmonary fibrosis. Interestingly, we observed that the mRNA expression of CSF1R decreased as a consequence of SC-43 treatment (Fig. 4D). Previous studies have identified STAT3 and NFκB as the key components regulating the transcription of CSF1R in neuro-inflammation [59]. STAT3 and NFκB also function as signal-dependent transcription factors of tissue-resident macrophages to drive the expression of inflammatory genes such as *CSF1R* in the CNS [59, 60]. Since SHP-1 is a negative regulator of the STAT3/NFκB pathway, the SHP-1 agonist SC-43 may thus downregulate *CSF1R* transcription in macrophages by enhancing SHP-1 activity.

NFκB has been shown to drive macrophage polarization toward M1 while STAT3 activation directs macrophages toward M2 fate [61]. On the other hand, STAT3 activation in fibroblasts has also been reported to promote fibroblast-to-myofibroblast differentiation, collagen deposition, and fibrosis [62–64]; hence, the possibility exists that the application of the SHP-1 agonist SC-43 may simultaneously target the STAT3/NFκB signaling in both the macrophages and fibroblasts, which are two of the most important cell populations for the pathogenesis of IPF, reshaping the immunofibrotic niche [65] and leading to the resolution of pulmonary fibrosis. The possibility that SC-43 may also target fibroblasts by inhibiting their STAT3 signaling is of great importance, since the anti-IPF medication nintedanib has been reported to suppress STAT3 and induce senolytic death in bleomycin-instilled mouse fibroblasts [66].

In summary, this study identifies SHP-1 as a druggable target for the treatment of IPF by employing the SHP-1 agonist SC-43. A pivotal role of macrophages triggering the progression of IPF has also been established, probably by secreting fibroblast-modulating mediators and inhibiting the fibroblast-to-myofibroblast transition. Our study paves the way for further development of SHP-1 agonists against IPF. Our strategy, by targeting macrophages for the treatment of pulmonary fibrosis, may also be applied in broader indications, as a high degree of similarity has been observed between the macrophage populations isolated from IPF patients and macrophages from COVID-19 patients [67].

## Supplementary information

Reproducibility checklist

Supplementary Information

## Data Availability

All data supporting the findings of this study are available with the article or from the corresponding author upon reasonable request.
