# Supplementary material for: Targeting pathogenic macrophages by the application of SHP-1 agonists reduces inflammation and alleviates pulmonary fibrosis
Source: Cell Death Dis. 2023 Jun 8;14(6):352. doi: 10.1038/s41419-023-05876-z (PMC10249559; doi:10.1038/s41419-023-05876-z)
Supplement: Supplementary file 2 — Supplementary Information [file 41419_2023_5876_MOESM2_ESM.pdf]

Table S1. Antibody panel used for single cell mass cytometry analysis

Antigen	Clone	Metal label	Mass	Source	Cat.No#	Staining concentration
CD45	30-F11	Y	89	Fluidigm	3089005B	0.5 µL/100 µL
CD4	RM4-5	Cd	112	Biolegend	100506	0.5 µg/mL
CCR2	QA18A56	In	113	Biolegend	160102	2 µg/mL
Gr1	RB6-8C5	Cd	114	Biolegend	108402	2 µg/mL
TCRb	H57-597	In	115	Biolegend	109202	4 µg/mL
TER119	Ter-119	La	139	Biolegend	116202	2 µg/mL
Ly6G	1A8	Pr	141	Fluidigm	3141008B	0.5 µL/100 µL
CD11b	M1/70	Nd	142	Biolegend	101202	3 µg/mL
CD11c	HL3	Nd	143	BD	553799	2 µg/mL
CD115	AFS98	Nd	144	Fluidigm	3144012B	1 µL/100 µL
CD172a	P84	Nd	145	Biolegend	144004	2 µg/mL
pSTAT1	4a	Sm	147	BD	custom order	2 µg/mL
CD103	2E7	Nd	148	Biolegend	121402	2 µg/mL
EpCAM	G8.8	Sm	149	Biolegend	118201	0.2 µg/mL
Ly6C	HK1.4	Eu	151	Biolegend	128002	0.5 µg/mL
CD3	145-2C11	Sm	152	Fluidigm	3152004B	0.1 µL/100 µL
CD274 (PD-L1)	10F.9G2	Eu	153	Fluidigm	3153016B	0.3 µL/100 µL
Siglec-H	551	Sm	154	Biolegend	129602	1 µg/mL
CD8a	53-6.7	Gd	155	Biolegend	100702	1 µg/mL
EOMES	Dan11mag	Gd	156	eBioscience	14-4875-82	2 µg/mL
pSTAT3	4/P-STAT3	Gd	158	Fluidigm	3158005A	1 µL/100 µL
CD47	Miap301	Tb	159	Biolegend	127502	2 µg/mL
CD206	C068C2	Gd	160	Biolegend	141702	1 µg/mL
TCRgd	GL3	Dy	162	Biolegend	118101	0.5 µg/mL
CD80	16-10A1	Dy	163	Biolegend	104735	0.5 µg/mL
Ikba	L35A5	Dy	164	Fluidigm	3164004A	1 µL/100 µL
CD31	390	Ho	165	Fluidigm	3165013B	1 µL/100 µL
CD19	6D5	Er	166	Fluidigm	3166015B	1 µL/100 µL
NKp46	29A1.4	Er	167	Biolegend	137602	1 µg/mL
PD-1	29F.1A12	Er	168	Biolegend	135202	1 µg/mL
F4/80	BM8	Tm	169	Biolegend	123103	2 µg/mL
CD137 (41BB)	17B5	Er	170	Biolegend	106114	2 µg/mL
CD64	X54-5/7.1	Yb	171	Biolegend	139301	6 µg/mL
CD86	GL-1	Yb	172	Biolegend	105002	0.5 µg/mL
Cleaved Caspase-3	D3E9	Yb	173	Cell Signaling	custom order	1 µg/mL
Siglec-F	E50-2440	Yb	174	BD	552125	0.5 µg/mL
CD44	IM7	Lu	175	BD	553131	0.2 µg/mL
B220/CD45R	RA3-6B2	Yb	176	Fluidigm	3176002B	0.1 µL/100 µL
I-A/I-E (MHCII)	M5/114.15.2	Bi	209	Biolegend	107602	0.5 µg/mL
DNA		Ir	191/193	Fluidigm	201192B	0.25 µM
Cisplatin Viability		Pt	195	Sigma-Aldrich	P4394-100MG	25 µM

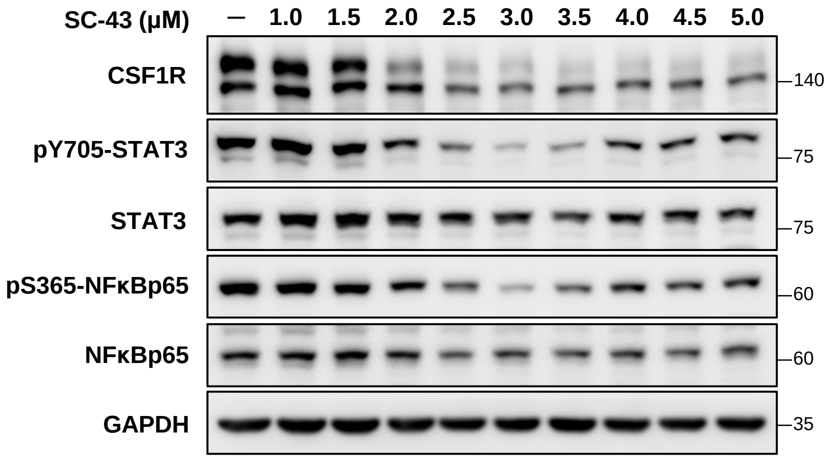

Figure S1. The effect of SC-43 on the expression of CSF1R and its downstream signaling. THP-1 cells were treated with indicated concentrations of SC-43 for 24h. DMSO was used in the control group. The levels of CSF1R, STAT3, NFκBp65 and β-actin were detected by Western blotting.

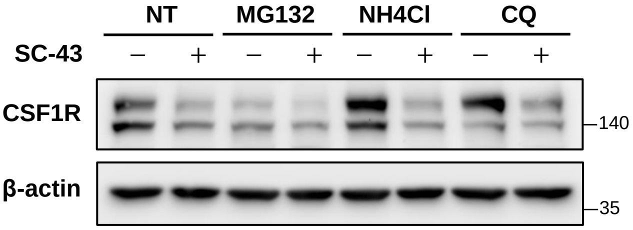

Figure S2. The effect of SC-43 on CSF1R levels following proteasomal, autophagic, or lysosomal inhibition. THP-1 cells were pretreated with or without 10μM MG132, 20mM ammonium chloride (NH4Cl) or 10μM chloroquine (CQ) in the presence or absence of 5 μM SC-43 for 24h (NH4Cl and CQ) or for 6h (MG132). DMSO was used in the control group. The levels of CSF1R and β-actin were detected by Western blotting.

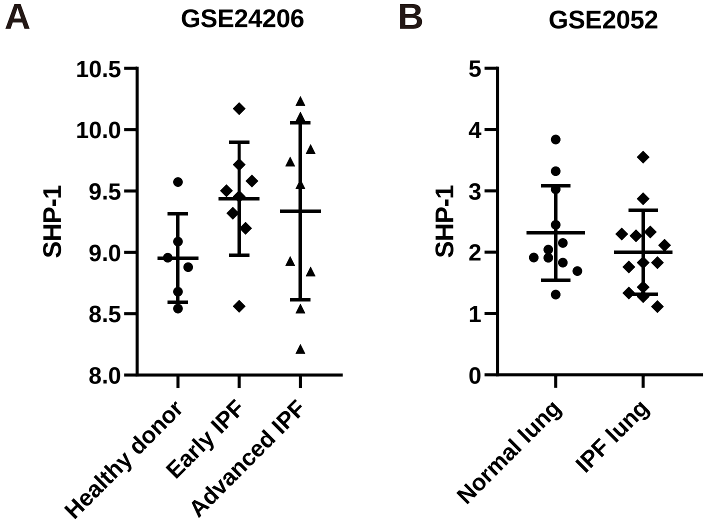

Figure S3. The expression levels of SHP-1 in normal and IPF lungs. The RNA expression data of normal and IPF lungs were extracted from (A) GSE24206 and (B) GSE2050 samples from the GEO database (https://www.ncbi.nlm.nih.gov/geo/). Each scatter dot plot was obtained for expression pattern analysis and generated using GraphPad Prism 8.0 (GraphPad Software, Inc.).

4C

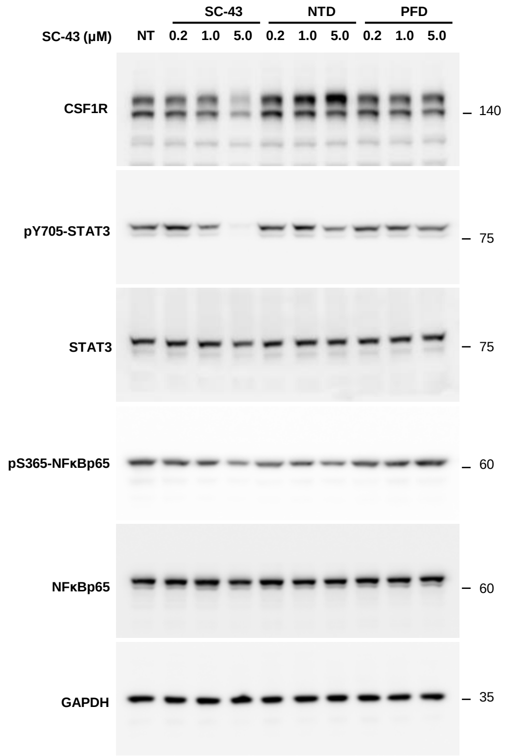

5A

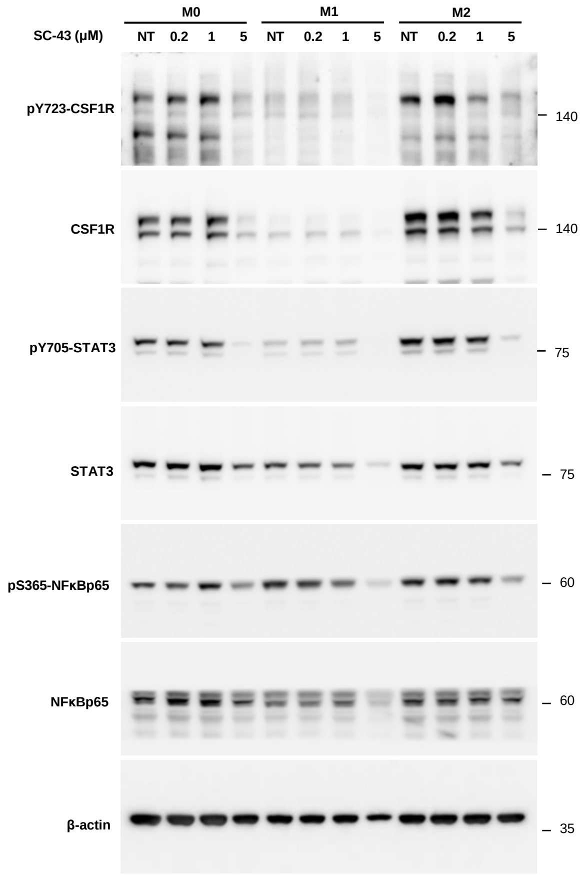

6C

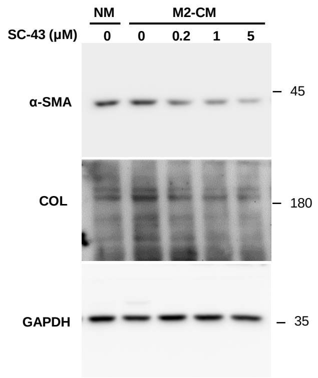

6D

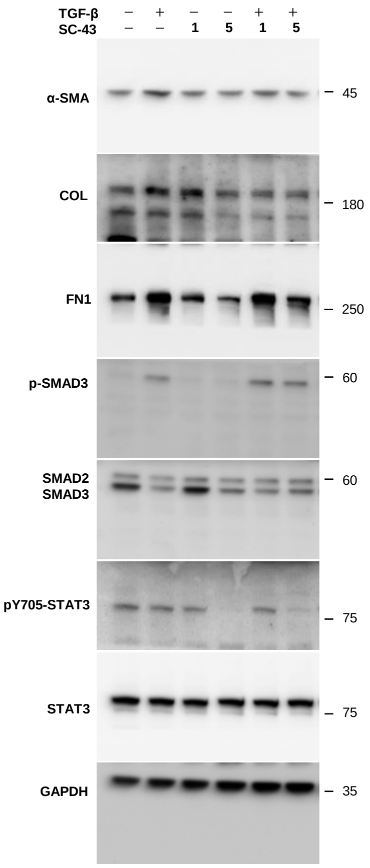

Figure S4. Original Western blots.
